# In Vitro Detection of Biologically Active Staphylococcal Enterotoxins Type B and C1 as an Alternative to In Vivo Testing

**DOI:** 10.3390/microorganisms14061383

**Published:** 2026-06-22

**Authors:** Reuven Rasooly, Naomi Balaban

**Affiliations:** 1 Emeritus, Foodborne Toxin Detection & Prevention Research Unit, Agricultural Research Service, USDA, Albany, CA 94710, USA; 2 Naomi Balaban, StaphOff Biotech Inc., Dudley, MA 01571, USA; nbalaban@staphoff.com

**Keywords:** SEB, SEC1, detection, staphylococcal enterotoxin, TCR Vβ3

## Abstract

*Staphylococcus aureus* is a major bacterial pathogen that can cause clinical infections and foodborne illnesses through the production of 25 exotoxin types. The most frequently implicated toxins in food poisoning outbreaks are Staphylococcal enterotoxins type A–E (SEA-SEE), which are the first enterotoxins discovered. While in vitro detection methods are available to identify the presence of enterotoxins, they cannot distinguish between biologically active and inactive forms of the toxins. Detection of biologically active enterotoxins currently relies on in vivo testing, using the emetic response in kittens or monkeys. Here, we show the development of an in vitro assay to detect the active forms of SEB, a potential biological warfare agent and leading cause of food poisoning, and SEC1, a frequent cause of staphylococcal food poisoning. The novel assay involves the implementation of a genetically engineered Jurkat T-cell line expressing TCR Vβ3, resulting in a dose response of IL-2 production when exposed to active toxin. We also show that at a concentration of 100 ng/mL, the biological activity of SEB is significantly decreased at temperatures over 70 °C, while pasteurization at 63 °C only slightly reduces the biological activity of the toxin. Our studies provide an alternative method to animal testing to determine the presence of active toxins and provide possible inactivation methods of the toxins.

## 1. Introduction

Foodborne diseases are one of the world’s leading health challenges, estimated to cause 420,000 deaths annually and economic losses in the range of $110 billion worldwide [1]. The most common causative molecules responsible for food poisoning are staphylococcal enterotoxins (SEs), which are produced by the bacterium *Staphylococcus aureus* and cause nearly a quarter of a million illnesses per year in the United States alone [2]. SEs are a group of twenty-five different types of potent toxins. They are classified into types based on the time of their discovery, classically designated by letters of the alphabet. The first enterotoxins discovered, SEA to SEE, are the most frequently implicated in food poisoning outbreaks.

SEs are divided into two groups. Group I includes SEA, SED, SEE, SEH, and SEI. Group II includes SEB, SEC, and SEG [3]. They are grouped based on amino acid sequence similarities and structural features. Enterotoxins SEB and SEC1 are highly similar in their structure, function, and biological properties. They belong to the same sequence homology group and exhibit significant cross-reactivity with antibodies [3]. They transit through the gastrointestinal tract, inducing emesis and triggering an excessive immune response by forming a bridge between the major histocompatibility complex (MHC) class II molecules of antigen-presenting cells (APC) and the variable region of the beta chain (Vβ) on the T-cell receptor [3,4,5]. This, in turn, causes CD4^+^ T-cells to proliferate and secrete cytokines [6,7]. When site-directed mutagenesis was used to inhibit the emetic activity of SEC, it resulted in the elimination of its superantigenic activities [8]. While APCs process antigens into peptide fragments for display on the cell surface via MHC class II, it was suggested that superantigens bypass this typical processing pathway. Instead of being broken down into peptides, superantigens bind directly to both MHC class II molecules and T-cell receptors, bypassing the common antigen processing and presentation steps.

It was demonstrated that when APCs were fixed by paraformaldehyde (which prevents any ongoing biochemical reactions in antigen-presenting cells while retaining the morphology of the antigenic sites), they could still bind and present an intact superantigen, thus bypassing the usual antigen processing. However, the level of cytokine secretion induced by superantigens that did not go through antigen processing was significantly lower than that induced by processed superantigens [9]. The ability to quickly identify the cause of an outbreak is very important to halt its spread and implement measures to prevent similar outbreaks from happening. The most common approaches for SEs detection are in vitro immunological methods, but these do not differentiate between inactive (harmless) and active forms of SEs, which are a threat to public health. Currently, only in vivo test methods using kittens and monkeys are available to determine if an enterotoxin is in its active (harmful) form. Clearly, these methods are cruel, costly, and lengthy.

To provide the food industry with a practical test of whether methods used for SEs inactivation are effective, we previously utilized their superantigenic activities that trigger splenocyte cells to secrete cytokines such as IFN-γ, TNF, and IL-2, rapidly upregulating the T-cell surface marker CD154. To avoid the need for using animal testing, we replaced the mouse splenocytes with the human CCRF-CEM T-cell line together with Raji B-cells as antigen-presenting cells (APCs) for the detection of biologically active SEA and demonstrated that within 2 h, SEA reduces surface T-cell receptor (TCR) Vβ9 in a dose-dependent manner over a 6-log range [9]. We also used the Jurkat T-cell line expressing the luciferase reporter gene and developed a bioluminescence-based assay for the detection and quantitation of biologically active SED and SEE, and demonstrated that SEE presented by Raji B-cells causes the internalization of the endogenous TCR Vβ8 in a dose-dependent manner over a 10-log range [10]. However, those in vitro assays cannot detect SEs that belong to Group II antigens, including SEC, whose toxicity has so far been only demonstrated in rhesus monkeys, where feeding of 5 µg SEC led to the production of emesis between 2 and 5 h after [11]. SEC plays an important role in the development of mastitis, affecting both humans and dairy cows [12,13]. It was shown that SEC directly induces mastitis in a mouse model through superantigenic activity that triggers the release of cytokines that intensify the inflammatory responses, leading to mammary tissue damage [12]. Other SE members that belong to the Group II antigens consist of SEB, which is attributed to the recent food poisoning outbreak in Turin, Italy [14], and the only enterotoxin classified by the U.S. Centers for Disease Control and Prevention as a category B agent of potential bioterrorism risk.

As an alternative to animal testing for measuring biologically active toxins that belong to Group II SEs, we examined the use of a genetically engineered Jurkat T-cell line expressing TCR Vβ3. We show here that incubation of SEB or SEC1 together with Raji B-cells as APCs and the genetically engineered Jurkat T-cell led to dose-dependent secretion of IL-2.

## 2. Materials and Methods

### 2.1. Chemicals and Reagents

Staphylococcal enterotoxins types B and C1 were obtained from Toxin Technology (Sarasota, FL, USA). The toxin concentrations were quantified by Toxin Technology, Inc., and their purity levels were determined to be greater than 95% using SDS-PAGE coupled with Coomassie blue staining. The toxins were dissolved in water before treatment.

Bromodeoxyuridine (5-bromo-2-deoxyuridine, BrdU), which mimics the natural nucleoside thymidine, was obtained from Calbiochem (San Diego, CA, USA).

PE-conjugated mouse anti-human TCR Vβ3 antibody (Cat# 566432) and a BD OptEIA ELISA kit for human IL-2 were obtained from BD Biosciences (San Jose, CA, USA). Lipopolysaccharide (LPS) was purchased from Sigma (St. Louis, MO, USA).

The viability assay stain eFluor 780 was purchased from Thermo Fisher (Waltham, MA, USA).

A genetically engineered T-cell leukemia Jurkat cell line stably expressing TCR Vβ3 was a generous gift from Dr. Balbino Alarcón, Autonomous University of Madrid, Spain. These engineered T-cells expressing TCR Vβ3 are now available through the cell culture facility of the University of California, Berkeley, located in 336 Barker Hall. The engineered T-cells were cultured in RPMI 1640 medium (Gibco, Carlsbad, CA, USA; 22400) supplemented with 10% fetal calf serum, with selection antibiotic G418 (Geneticin) at a concentration of 0.1 mg/mL. Cell culture medium of 500 mL also contained 2 mL of 100X Antibiotic-Antimycotic (Gibco/Thermo Fisher, Waltham, MA, USA) and 2.5 mL of 100X (200 mM) L-Glutamine (Gibco/Thermo Fisher, Waltham, MA, USA).

Burkitt’s lymphoma Raji B-cell line (ATCC number CCL-86) was purchased from the American Type Culture Collection (ATCC, Rockville, MD, USA). The Raji B-cell line was cultured in RPMI 1640 medium (Gibco, 22400) supplemented with 100 units/mL penicillin, 100 µg/mL streptomycin (Gibco, Carlsbad, CA, USA; 15140-122), 10% fetal calf serum, 100 nM sodium pyruvate (Invitrogen, Waltham, MA, USA; #11360), and 1% MEM non-essential amino acids (Invitrogen, Waltham, MA, USA; #11140).

### 2.2. Isolation of Splenocytes

Mice were euthanized by rapid cervical dislocation. Splenocytes were collected by needle and syringe disruption of the spleens that were aseptically removed from 9-month-old female C57BL/6 mice. Disruption of the spleens was performed in Russ-10 cell culture medium prepared from RPMI 1640 medium without glutamine (Gibco, Carlsbad, CA, USA) supplemented with 10% fetal bovine serum (HyClone, Logan, UT, USA), 2 mM glutamine (Gibco), 1 mM sodium pyruvate (Gibco), 1× MEM NEAA, 1× antibiotic-antimycotic (Gibco; containing penicillin, streptomycin, and fungizone), 50 mM β-mercaptoethanol (Sigma, St. Louis, MO, USA) and 5 mL of non-essential amino acid mix (Gibco). Harvested splenocytes were centrifuged at 200× *g* for 10 min at 4 °C, and red blood cells were removed in lysis buffer consisting of 150 mM NH_4_Cl, 100 µM Na_2_EDTA, and 10 mM KHCO_3_. The cells were centrifuged a second time and finally resuspended in Russ-10 medium.

Viable cell counts were performed using a hemocytometer based on the exclusion of trypan blue, which differentiates between live and dead cells.

The splenocytes and cell lines were maintained in an incubator kept at 37 °C under a humidified atmosphere containing 5% CO_2_.

### 2.3. Assess Cell Proliferation by BrdU Incorporation

Splenocyte cells were placed in 96-well plates at a concentration of 1 × 10^6^/mL in a total volume of 0.2 mL in Russ-10 medium. Cells were treated in triplicate wells with increasing concentrations of SEB (1 ng/mL to 20 μg/mL). Negative control was the addition of media only (no SEB). Positive control was the addition of 1 μg/mL LPS. Following incubation at 37 °C in a 5% CO_2_ incubator for 72 h, cell proliferation was measured by adding the analog of thymidine, bromodeoxyuridine (5-bromo-2-deoxyuridine, BrdU) to each well. After 4 h, cells were fixed, permeabilized, and DNA denatured according to the manufacturer’s instructions (Calbiochem, San Diego, CA, USA). Briefly, diluted BrdU was added to the cells and incubated at 37 °C for 4 h. The unincorporated BrdU was removed by centrifugation (spinning the plates at 200× *g* for 10 min). Denaturing fixative reagent (200 µL/well) was added and incubated for 30 min at room temperature, and then poured out. The anti-BrdU antibody, conjugated to horseradish peroxidase, was then added (100 µL/well) and incubated for 90 min at room temperature. The wells were washed three times with PBS, the substrate TMB (3,3′,5,5′-tetramethylbenzidine) was added at 100 µL/well, and incubated for 5 to 30 min until the development of color was sufficient. The spectroscopic measurements at absorbances of 450 and 620 nm were made to assess T-cell proliferation.

### 2.4. Cytometric Bead Array to Simultaneously Quantify Multiple Cytokines from a Single Sample

Cytometric bead array was used to quantify multiple cytokine secretion to the cell culture medium containing 1 × 10^6^ cells per mL of modified Jurkat cells and 5 × 10^5^ cells per mL of Raji B-cells and with SEB at concentrations of 0, 0.001, 0.01, 0.1, 1, 100, 1000, 10,000, and 100,000 ng/mL. Cells were incubated at 37 °C for 48 h, removed, and analyzed using the cytometric bead array human Th1/Th2/Th17 cytokine kit from BD Bioscience according to the manufacturer’s instructions. A flow cytometer (BD Bioscience FACSAria Fusion) was used to collect bead data, and FlowJo software version 10 application developed by BD Biosciences was used to analyze the data. An ELISA assay was used (in triplicate) to confirm that SEB induces IL-2 secretion in a dose-dependent manner. Specific methods are described below.

### 2.5. Flow Cytometry to Confirm That the Modified T-Cell Line Expresses TCR Vβ3

Cells were prepared for flow cytometry by removing the growth medium and washing the cells twice with PBS by centrifugation at 200× *g* for 10 min, followed by removal of the supernatant. Cells were resuspended in PBS, stained at 4 °C in the dark for 30 min (see below), and then washed twice to remove unbound stain. The cells were resuspended in PBS, and flow cytometry was carried out using an instrument from BD Biosciences, model FACSAria Fusion cytometer (San Jose, CA, USA). TCR Vβ3 levels were measured by staining with PE fluorescence-labeled anti-Vβ3 mAb (BD Pharmingen) and were quantified in the 585/42 nm bandpass channel. Dead cells were identified and excluded from analysis by staining with the dead cell stain eFluor 780 (Thermo Fisher, Waltham, MA, USA) and their fluorescence measured in the 780/60 nm bandpass channel. Other contaminants and cell debris were excluded from analysis by gating the cell population based on forward and side light scattering. To accurately identify TCR Vβ3 in the modified T-cells, data from 10,000 cells were analyzed using BD Biosciences’ FlowJo software.

### 2.6. Measuring the Levels of IL-2 Secretion in Response to Exposure to SEB or SEC1

In a clear 96-well plate, 50 µL of 2 × 10^6^/mL cell suspension of the engineered T-cells, 25 µL of a 2 × 10^6^/mL cell suspension of Raji B-cells, and 25 µL of SEB or SEC1 at four times the final target concentration were combined in Raji’s cell culture medium and incubated at 37 °C for up to three days. The supernatants were then harvested by centrifugation at RCF of 200× *g* for 10 min and were further tested for IL-2 secretion by ELISA according to the manufacturer’s instructions (BD Bioscience, San Jose, CA, USA; OptEIA Human ELISA).

## 3. Results

### 3.1. Confirmation That the Modified T-Cell Line Used in This Study Expresses the Human TCR Vβ3

**Figure 1:**
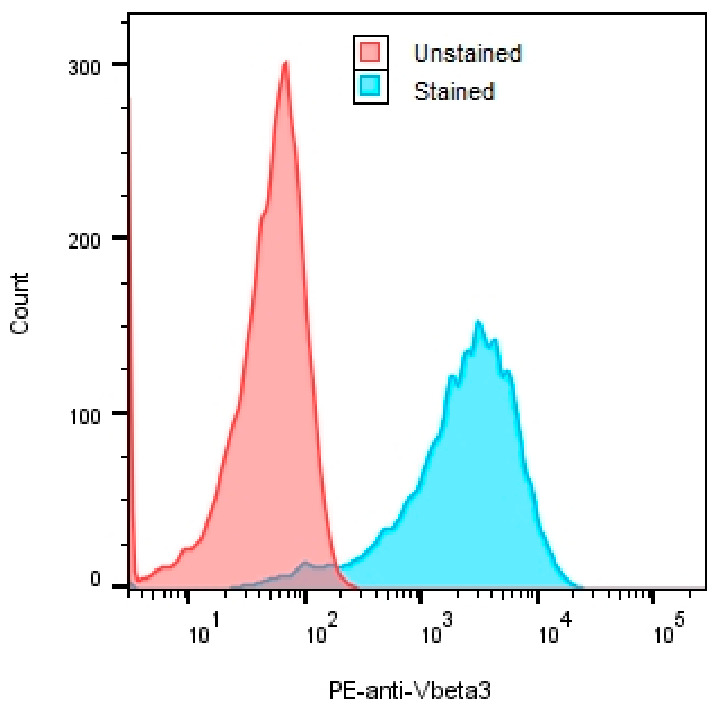
A genetically modified Jurkat cell line that expresses TCR Vβ3 was stained with PE-labeled antibody to TCR Vβ3 and compared with control unstained cells. The flow cytometric histogram *x*-axis represents the signal intensity of cell-associated PE fluorescence on a logarithmic scale, and the histogram *y*-axis (count) represents the number of modified Jurkat cells at a given intensity level.

To evaluate if Jurkat T-cell line that is genetically engineered to express TCR Vβ3 can be used to measure biologically active SEB, we first confirmed that the cell line used in this study expresses TCR Vβ3 by staining the cells with PE-conjugated anti-TCR Vβ3 followed by flow cytometry analysis. The histogram results (Figure 1) show the binding of PE-conjugated anti-Vβ3 monoclonal antibody to the Vβ3 region of the TCR and visualize the intensity and frequency distribution of Vβ3 in modified T-cells. The *x*-axis represents the fluorescence intensity emitted by the phycoerythrin fluorescent dye attached to Vβ3, which indicates the amount of Vβ3 present in the cells. The *y*-axis indicates the number of modified Jurkat T-cells expressing specific levels of Vβ3.

### 3.2. Ex Vivo and In Vitro Cell-Based Assay for Detection of Biologically Active SEB

**Figure 2:**
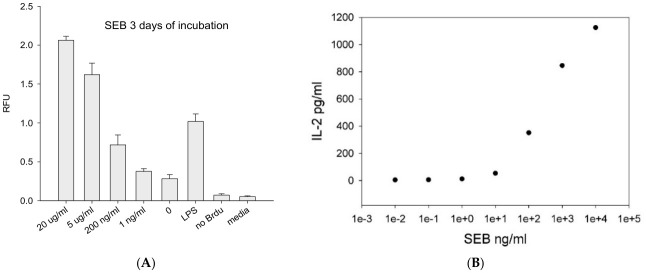
Detection of SEB by ex vivo mouse splenocyte proliferation assay and by in vitro assay based on the secreted IL-2 from combined B-cells and modified T-cells. The combined T-cells and B-cells or mouse splenocytes were spiked with SEB and 1 μg/mL of lipopolysaccharide (LPS) as a positive control to assess the rate of the proliferation responses. (**A**). After incubation for 3 days, the relative concentrations of the secreted IL-2 were determined by flow cytometry, and newly synthesized DNA was measured by ELISA (**B**). Error bars represent standard errors.

**Figure 3:**
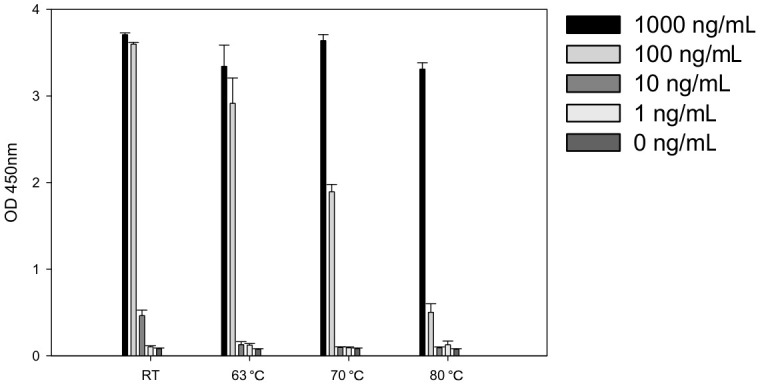
Thermal treatment reduces the biological activity of SEB. PBS was spiked with increasing concentrations of SEB and heated at 63 °C, 70 °C, and 80 °C for 30 min. Control samples were spiked with PBS containing no SEB. After incubation for 72 h, secretion of IL-2 from the modified T-cells was determined by ELISA. Error bars represent standard errors.

To evaluate the ability of the *ex vitro* cell-based assay to quantify SEB, the effect of various concentrations of SEB, ranging from 1 ng/mL to 20 μg/mL, on splenocyte proliferation was measured. Newly synthesized DNA was measured by adding BrdU (an indication of cell proliferation), followed by spectroscopic measurements. As shown in Figure 2A, the amount of newly synthesized DNA measured by BrdU incorporation is proportional to SEB concentration. In terms of sensitivity, this splenocyte proliferation assay enables the detection of 200 ng/mL of SEB, which is 60 times more sensitive than the monkey bioassay in which vomiting occurs in 50% of monkeys on administration of 12 μg [15,16]. As shown in Figure 3, when treatment is performed at room temperature (RT), the two assays are less sensitive than the in vitro assay, which has a limit of detection of 10 ng/mL. To evaluate if a genetically modified Jurkat cell line that expresses TCR Vβ3 in conjunction with Raji B-cells can be used to detect biologically active SEB, decreasing concentrations of SEB were incubated for three days with the combined modified Jurkat T-cells and Raji B-cells lines or with mouse splenocyte cells. As demonstrated in Figure 2, both ex vivo and in vitro assays can be used to quantify biologically active SEB in a dose-dependent manner. The in vitro assay is 20 times more sensitive than the ex vivo assay. Specifically, Figure 2A shows that >200 ng/mL SEB significantly (*p* < 0.05) induces mouse splenocyte proliferation as compared to control untreated cells (as demonstrated by BrdU incorporation into newly synthesized DNA). Figure 2B illustrates the successful use of a genetically engineered TCR Vβ3 T-cell line combined with Raji B-cell lines to quantify biologically active SEB through interleukin 2 (IL-2) secretion.

### 3.3. Thermal Treatment Reduces the Biological Activity of SEB

To confirm the flow cytometry results and to examine the effect of heat treatment on the toxin, we used a more convenient ELISA assay, which does not require costly equipment and skilled operators. PBS was spiked with 1 µg/mL, 100 ng/mL, 10 ng/mL, 1 ng/mL, or 0 ng/mL of SEB and was then exposed to heat at 63 °C, 70 °C, and 80 °C for 30 min. Those were then applied to the T-cells that had been genetically altered to express Vβ3 TCR combined with a Raji B-cell line that presents the SEB-MHC class II, leading to IL-2 secretion.

As shown in Figure 3, the effectiveness of thermal inactivation is influenced by both the temperature and the initial SEB concentration. At a concentration of 100 ng/mL, SEB biological activity was significantly (*p* < 0.05) decreased in a temperature-dependent manner, particularly when exposed to temperatures of 70 °C and 80 °C. The higher the temperature, the greater the loss of SEB biological activity. The loss of SEB biological activity is more noticeable at a concentration of 100 ng/mL than 1000 ng/mL because at lower concentrations, the T-cell receptor Vβ3 is not as saturated with SEB, so the increased inactivation due to higher temperature has a greater impact on the direct binding rate of Vβ3 to the denatured SEB that lost some of its three-dimensional structure.

### 3.4. Detection of Active SEC1 Using IL-2 Secretion by the T-Cell Line Expressing TCR Vβ3

**Figure 4:**
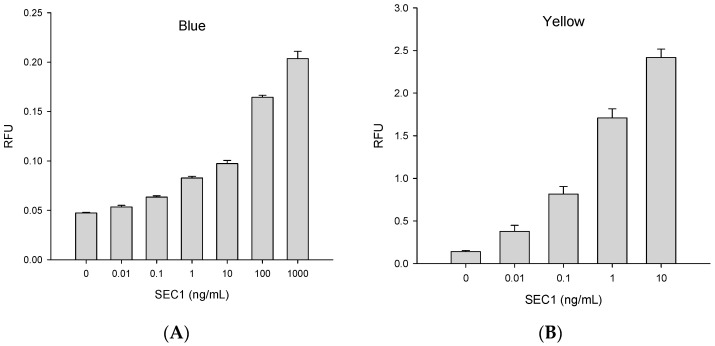
Detection of SEC1 by in vitro assay based on the secreted IL-2 from combined B-cells and modified T-cells. After incubation for 3 days, the relative concentrations of the secreted IL-2 were determined by ELISA. Error bars represent standard errors. (**A**) Blue: color development before the addition of the stop solution. (**B**) Yellow: color development after addition of stop solution. Error bars represent standard errors.

To evaluate if the modified cell line method can be used to detect other members of Group II antigens, we used SEC1. Increasing concentrations of SEC1 added to the genetically engineered Jurkat T-cell line and Raji B-cell line, which would present SEC1 to the Vβ3 TCR, led to secretion of IL-2 that can be detected by a sandwich ELISA. In this method, the conjugated horseradish peroxidase (HRP) antibody binds to the secreted IL-2, then catalyzes the oxidation of the TMB (3,3′,5,5′-tetramethylbenzidine) substrate that forms a weak, blue-colored signal (that represents the active, developing phase of the test), which is directly proportional to the amount of IL-2 present in the sample (Figure 4A). This activity assay, even with a weak signal, is more sensitive than the in vivo monkey bioassays, where feeding of 3 µg SEC1 causes an emesis response in 50% of the animals. As shown in Figure 4B, to significantly enhance the signal by more than ten times, 2 N sulfuric acid was added, which converts the blue product to a final yellow derivative that has a maximum absorbance at 450 nm. Consequently, at a concentration of 1000 ng/mL, the signal is too strong and exceeds the instrument’s detection range, leading to an “overflow” indication instead of a numerical value. Previously, we showed that SEB and SEC exhibited no response to the combined APC and Jurkat T-cell line expressing the endogenous T-cell receptor Vβ8. Here, as shown in Figure 4, exposure of SEC1 to the combined APC and the genetically engineered Jurkat T-cell line expressing TCR Vβ3 induces differential IL-2 secretion over a 4-log range, without requiring multiple sample dilutions. The limit of detection of biologically active SEC1 is 10 pg/mL, which is 3 × 10^6^ times more sensitive than the monkey bioassay, in which vomiting occurs in 50% of monkeys upon administration of 3 μg toxin [15,16]. The limit of detection was significant (*p* < 0.05) from 0.01ng/mL SEC1 and higher.

### 3.5. SEB at Concentrations of 10 µg/mL and 1 µg/mL Induces a Dose-Dependent and Rapid Internalization of TCR Vβ3, While SEC1 Has No Such Effect

**Figure 5:**
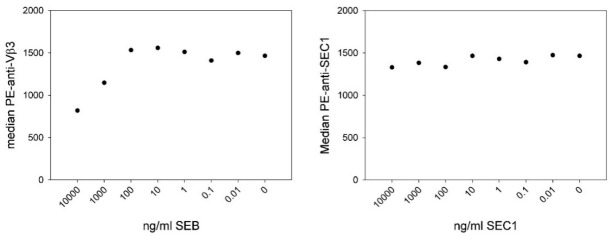
SEB induces rapid internalization of TCR Vβ3 in a dose-dependent manner. Jurkat T-cells and Raji B-cells were mixed and co-incubated with increasing concentrations of SEB for 2 h. Mean fluorescence intensity for TCR Vβ3 was measured by flow cytometry after the stimulated cells were stained with PE-conjugated anti-Vβ3 mAb.

Using flow cytometry, we tested if genetically modified T-cells expressing TCR Vβ3 can be used for the rapid detection of biologically active members that belong to the Group II antigens SEB and SEC1. As illustrated in Figure 5, following a two-hour co-incubation of biologically active SEB or SEC1 with the mixed Raji B-cells and the genetically modified Jurkat T-cell line, only at high concentrations of SEB is there a dose-dependent reduction in TCR Vβ3 protein detection in the T-cell population, while the lower-affinity ligand SEC1 has no such effect. These results suggest that strong internalization of TCR Vβ3 from the cell surface within two hours is not a precondition step necessary to trigger cytokine secretion to further regulate the immune response.

## 4. Discussion

Current techniques for the detection and quantification of active Staphylococcal enterotoxins involve animal studies, such as measuring the emetic response of kittens or monkeys. Those bioassays require many animals and raise ethical, moral, and philosophical issues. In addition, animal-based assays are often insensitive and may yield inconsistent results because of animal variability. Methods such as liquid chromatography-mass spectrometry and enzyme-linked immunosorbent assays (ELISAs) have been developed for several enterotoxins. However, these methods cannot differentiate between the inactive form of the toxin that is harmless and the active form of SEs that poses public health threats. It was suggested that measuring SEs’ superantigenic activity can predict the toxins’ activity without animal testing. But when site-directed mutagenesis was used to inhibit SEC emetic activity, it also eliminated T-cell activation [8]. We developed in vitro methods to test whether SE inactivation methods are effective and to ensure that the food supply is safe. We have utilized different in vitro immunological methods to replace the in vivo emetic bioassays for the detection of SE types A, B, C, D, and E, which are the most frequently implicated in food poisoning outbreaks. We used Raji B-cells as antigen-presenting cells with the human CCRF-CEM T-cell line that expresses the T-cell receptor Vβ9 for the detection of SEA [9]. For quantitation analysis of biologically active SED and SEE, we utilized the Jurkat T-cell line expressing the endogenous T-cell receptor Vβ8 together with Raji B-cells [10]. However, these assays cannot detect SEs that belong to Group II antigens, such as SEC, which plays an important role in the development of mastitis through proinflammatory cytokine release, inflammatory responses that subsequently induce mammary tissue damage in humans, goats, and cows [12]. The above assays also fail to detect SEB, another member of Group II antigens, which caused the recent food poisoning outbreak in Turin, Italy [14]. Our goal in this paper was to develop in vitro methods for the detection of active SEB and SEC, using data that demonstrates that SEB activates T-cells bearing Vβ3 [17].

Here, we demonstrate the successful use of a genetically engineered Jurkat T-cell line, in which the endogenous TCR Vβ8 was replaced with TCR Vβ3, for measuring the effect of biologically active SEB and SEC1. We utilized the superantigenic activity of SEB by adding to mouse splenocytes a thymidine analog that incorporates into newly synthesized DNA, and demonstrated here that the effect of SEB on spleen cell proliferation is like SEB’s effects on IL-2 secretion by the genetically engineered T-cell line. It was shown that levels of SEs expected to be found in food involved in food poisoning outbreaks are in the range of 0.5–10 μg per 100 mL or 100 g [18]. This toxin level is reached when the bacterial population exceeds 100,000 *S. aureus* in a gram of food [19]. Figure 3 and Figure 4 show that our assay’s ability to detect SEB and SEC1 is below the food contamination levels. The limit of detection of biologically active SEC1 with the in vitro assay is 10 pg/mL, which is 3 × 10^6^ times more sensitive than the monkey bioassay in which vomiting occurs in 50% of monkeys on administration of 3 μg [15,16]. Figure 2A shows that the splenocyte proliferation assay enables detection of 200 ng/mL of SEB, which is 60 times more sensitive than the monkey bioassay in which vomiting occurs in 50% of monkeys on administration of 12 μg SEB [15,16]. As shown in Figure 3, the ex vivo assay is less sensitive than the in vitro assay, which has a limit of detection of 10 ng/mL for SEB. We also tested if thermal processing, which is used to destroy spoilage microorganisms such as the bacterium *Staphylococcus aureus*, also reduces the biological activity of enterotoxins that had already been secreted. As shown in Figure 3, we utilized our in vitro method that can distinguish between biologically active and reduced activity of Group II antigens to assess the effectiveness of heat treatment on SEB. The results show that increasing temperatures cause a reduction in SEB activity in a temperature-dependent manner. The data presented here suggest that heat treatment causes SEB to denature and lose part of its three-dimensional structure, which consequently reduces its ability to bind T-cell receptor Vβ3, as detected through reduced release of IL-2. The effect of heat inactivation on SEB is more noticeable at a concentration of 100 ng/mL than at 1000 ng/mL, because at lower concentrations, the T-cell receptor Vβ3 is not as saturated with SEB, so the increase in inactivation due to higher temperature has a greater impact on the direct binding rate of SEB to Vβ3. Here, we used a purified SEB, suggesting that food-made SEB is more thermostable because it can bind and be sequestered by other molecules within the food matrix. Our flow cytometry analysis shows that two hours after SEB interaction with APC and the T-cell line, the TCR Vβ3 protein level was reduced in a dose-dependent manner, while SEC1 had no such effect. These findings suggest that (1) internalization of TCR Vβ3 can be used for quicker SEB detection. (2) Strong internalization of TCR Vβ3 from the cell surface is not a necessary precondition step to trigger IL-2 secretion, which in turn plays a significant role in regulating the immune response through influencing the activity of various immune cells.

Our data strongly suggest that a genetically engineered T-cell line expressing TCR Vβ3 together with Raji B-cells can effectively substitute for animal models to measure SEB and SEC1 activity. This approach can be used as an important tool to ensure food safety.

## Data Availability

The original contributions presented in this study are included in the article. Further inquiries can be directed to the corresponding author.
